# Supplementary material for: Emerin Represses STAT3 Signaling through Nuclear Membrane-Based Spatial Control
Source: Int J Mol Sci. 2021 Jun 22;22(13):6669. doi: 10.3390/ijms22136669 (PMC8269395; doi:10.3390/ijms22136669)

Supplementary Table. 1. HeLa cells were transiently transfected with an emerin expression vector and subjected to transcriptional profiling. Data from two independent experiments were normalized to GAPDH expression and are presented as EMD(emerin)/CTL(control) ratios.

Supplementary Figure 1. (A) C2C12 cells were treated with siRNA (100 nM) against *Emerin,* or *control* for 48 hours in 6 well plates. After 48 hours, cells were treated with differentiation media (DM) for 5 days. The total RNA was isolated and subjected to a qRT-PCR analysis. Data were normalized to *β-Actin*. The results represent the mean ±S.D. of three independent experiments performed in triplicate. *, $P < 0.05$, ** $P < 0.001$. (B) C2C12 cells were treated with siRNA (100 nM) against *Emerin,* or *control* for 48 hours in 6 well plates. After 48 hours, cells were treated with differentiation media (DM) for 5 days. Immunocytochemistry image stained with anti-Myosin Heavy Chain (MYHC) antibody from emerin-depleted C2C12 cells at day 5 of differentiation. DAPI (blue) was used to visualize nucleus. Scale bar=50 μm

Supplementary Table 1. Transcription factor profiling analysis with emerin transfected HeLa cells

Rank	Gene	EMD/CTL	Rank	Gene	EMD/CTL	Rank	Gene	EMD/CTL
1	E2F6	0.01	29	PPARA	0.18	57	DR1	0.53
2	ELK1	0.02	30	RELA	0.21	58	HAND2	0.54
3	FOXA2	0.02	31	b-Catenin	0.22	59	NFATC4	0.56
4	STAT1	0.02	32	SMAD5	0.22	60	ATF3	0.58
5	TFAP2A	0.02	33	TBP	0.23	61	ETS2	0.58
6	POU2AF1	0.03	34	NR3C1	0.24	62	HNF4A	0.59
7	TGIF1	0.03	35	CREB1	0.27	63	NFKB1	0.6
8	NFYB	0.04	36	RB1	0.27	64	ATF2	0.65
9	PAX6	0.04	37	g-CEBP	0.29	65	SMAD1	0.68
10	STAT5B	0.04	38	TCF7L2	0.29	66	GATA2	0.74
11	JUN	0.05	39	JUND	0.3	67	ARNT	0.76
12	FOXO1	0.06	40	GATA1	0.32	68	GTF2B	0.76
13	MYOD1	0.06	41	STAT2	0.32	69	NFATC1	0.77
14	STAT3	0.06	42	MAX	0.36	70	a-CEBP	0.81
15	JUNB	0.09	43	ATF4	0.37	71	GATA3	0.82
16	E2F1	0.1	44	IRF1	0.37	72	MEF2A	0.82
17	SP3	0.1	45	NFATC3	0.37	73	MEF2C	0.83
18	YY1	0.11	46	SP1	0.37	74	HAND1	0.84
19	CREBBP	0.12	47	NFATC2	0.38	75	AR	0.85
20	NFAT5	0.12	48	FOXG1	0.45	76	ATF1	0.86
21	STAT6	0.12	49	HSF1	0.46	77	FOS	0.86
22	PPARG	0.13	50	ETS1	0.47	78	b-CEBP	0.89
23	SMAD4	0.13	51	HDAC1	0.48	79	GTF2F1	0.95
24	HOXA5	0.14	52	STAT4	0.48	80	MYB	0.97
25	MYC	0.15	53	TP53	0.48	81	EGR1	0.98
26	STAT5A	0.16	54	ESR1	0.49	82	HIF1A	1.17
27	SMAD9	0.17	55	MYF5	0.5	83	RELB	1.53
28	HNF1A	0.18	56	REL	0.52	84	ID1	1.83

STAT signal related genes (Red) **Muscle development related genes (Blue)**

Supplementary Figure 1.

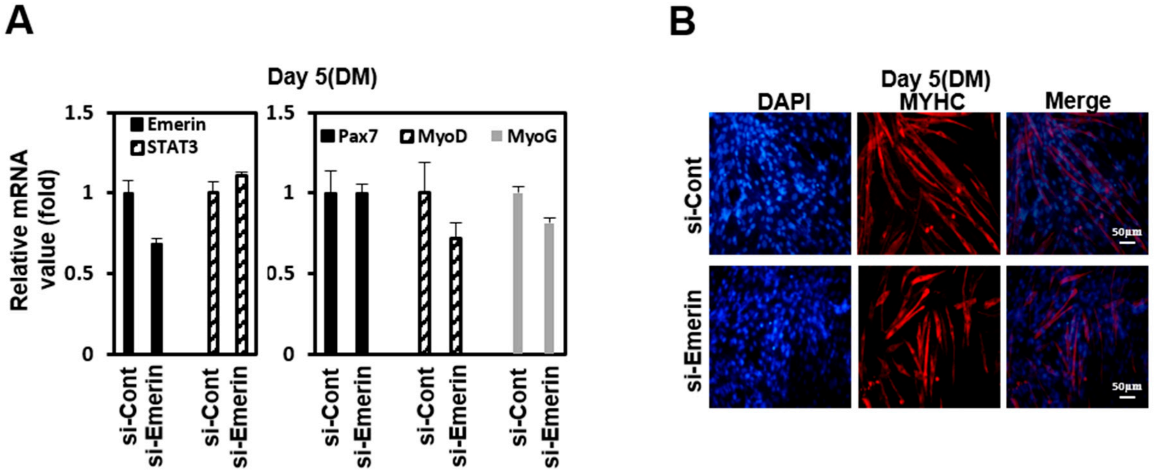

Supplement: Supplementary file 1 [file ijms-22-06669-s001.zip › ijms-1247976-supplementary.pdf]
